# Editorial for the Special Issue Titled “Advancements in Food Gelation: Exploring Mechanisms and Applications”

**DOI:** 10.3390/gels11080576

**Published:** 2025-07-24

**Authors:** Zhouyi Xiong, Xiaohu Luo, Qun Huang, Noman Walayat

**Affiliations:** 1 School of Life and Health Technology, Dongguan University of Technology, Dongguan 523808, China; 2 Dongguan Food Industry Science and Technology Innovation Center, Dongguan 523808, China; 3 College of Food and Pharmaceutical Science, Ningbo University, Ningbo 315000, China; 4 Department of Food Science and Engineering, School of Public Health, Guizhou Medical University, Guiyang 550025, China; 5 Centro Tecnológico de la Carne de Galicia, Parque Tecnológico de Galicia, San Cibrán das Viñas, Rúa Galicia N 4, 32900 Ourense, Spain; noman.rai66@gmail.com

Food gelation has emerged as a dynamic and multidisciplinary research field, bridging colloidal science, processing innovation, material engineering, and nutritional functionality [1,2]. Gels are critical to both the sensory and structural characteristics of food products and also serve as vehicles for the delivery of bioactives, textural design, and shelf life enhancement [3,4]. With the growing demand for personalized, sustainable, and functional foods, advancements in food gelation offer promising directions for novel food development and processing technologies [5].

This Special Issue, entitled “Advancements in Food Gelation: Exploring Mechanisms and Applications”, gathers nine contributions that showcase recent progress in gelation mechanisms, structure–function relationships, innovative materials, and applied technologies. These papers collectively highlight the versatility of gel systems—from starch and protein networks to hybrid hydrogel films and emulsion gels—demonstrating their significance across diverse food categories and processing environments.

Yang et al. investigated the effect of heat–moisture treatment on black soybean–wheat composite flours and cookies. Their study showed that this simple pre-treatment significantly improved nutritional quality and reduced starch digestibility, without compromising texture. In a related work, Yang et al. further explored the role of potato flour substitution in wheat flour systems, detailing its impact on viscosity, dough rheology, and gluten network formation.

Protein-based gels have shown unique potential in food preservation. Zhang et al. developed ovalbumin gel nanoparticles for encapsulating carvacrol, demonstrating improved heat/salt stability and sustained release for pork shelf life extension. Similarly, Zadeike et al. encapsulated Lacticaseibacillus paracasei in alginate–chitosan microgels, enabling enhanced viability through sourdough fermentation and baking processes and consequently offering new strategies for probiotic breadmaking.

Starch modification continues to be an important method for tailoring gel functionality. Li et al. acid-hydrolyzed quinoa starch to generate nanocrystals that stabilize high-internal-phase emulsion gels (HIPE gels), revealing the potential of compact crystalline structures in emulsified gel systems. Nguyen et al. applied ultrasound to modify durian starch, enhancing its thermal stability and functional properties for use in gels and films.

Applications of hybrid gel systems were also explored in this Special Issue. Xue and Huang prepared flaxseed oil/beeswax oleogels and successfully incorporated them into sodium alginate films to improve barrier properties and preserve marine fish. In another contribution, Xue et al. developed a composite edible film using perilla essential oil, grape seed extract, chitosan, and gelatin, which significantly improved grass carp freshness during storage.

In the field of structural design, Dushina et al. investigated 3D-printable gel inks enriched with lupine callus tissue, providing valuable insights into the effects of plant-derived biomass on rheological and mechanical properties, facilitating novel approaches in personalized nutrition and food fabrication.

Collectively, these contributions advance our understanding of food gelation from both mechanistic- and application-oriented perspectives. They reflect the expanding scope of gel science not only in conventional foods but also in smart food design, active packaging, and health-oriented formulations.

We extend our sincere appreciation to all authors and reviewers for their valuable contributions and insightful comments, which have greatly enriched the quality of this Special Issue. We also thank the editorial team of *Gels* for their professional support. We hope this collection will serve as a useful reference and inspiration for researchers and professionals working at the interface of food structure, functionality, and innovation.

## References

[1] Ashfaq A., Jahan K., Islam R.U., Younis K. (2022). Protein-based functional colloids and their potential applications in food: A review. LWT.

[2] Wijaya W., Patel A.R., Setiowati A.D., Van der Meeren P. (2017). Functional colloids from proteins and polysaccharides for food applications. Trends Food Sci. Technol..

[3] Hu B., Zhang Y., Han L., Zhao Y., Zhang C., Cao J., Yang J., Fang Y. (2025). Large deformation of food gels: Influencing factors, theories, models, and applications—A review. Food Res. Int..

[4] Huang S., Zhang Y., Chen Q., Liu Y., Lu L., Arain M.M., Pan S., Liu F. (2025). Pectin based gels and their advanced application in food: From hydrogel to emulsion gel. Food Hydrocoll..

[5] Fu K., Wang H., Pan T., Cai Z., Yang Z., Liu D., Wang W. (2024). Gel-forming polysaccharides of traditional gel-like foods: Sources, structure, gelling mechanism, and advanced applications. Food Res. Int..

